# Histatins, proangiogenic molecules with therapeutic implications in regenerative medicine

**DOI:** 10.1016/j.isci.2024.111309

**Published:** 2024-11-01

**Authors:** Héctor Tapia, Pedro Torres, Carlos Mateluna, Mónica Cáceres, Vicente A. Torres

**Affiliations:** 1 Institute for Research in Dental Sciences, Faculty of Dentistry, Universidad de Chile, Santiago, Chile; 2 Advanced Center for Chronic Diseases (ACCDiS), Universidad de Chile, Santiago, Chile; 3 Millennium Institute on Immunology and Immunotherapy (IMII), Santiago, Chile; 4 Institute of Biomedical Sciences (ICBM), Universidad de Chile, Santiago, Chile

**Keywords:** Health sciences, Medicine, Natural sciences, Biological sciences, Physiology

## Abstract

Recent studies show that a group of salivary peptides, collectively known as histatins, are potent inducers of wound healing in both soft and hard tissues. Among these molecules, histatin-1 stands out for its ability to stimulate the repair of skin, oral mucosal, and osseous tissue. Remarkably, all these effects are associated with the capacity of histatin-1 to promote angiogenesis via inducing endothelial cell adhesion, migration, and signaling. These findings have opened new opportunities in the field of regenerative medicine, leading to an increasing number of articles and patents proposing therapeutic uses of histatin-1. However, this scenario raises a relevant concern regarding the appropriate use of these molecules, since, unlike the mode of action, little is known about the molecular mechanism by which they promote angiogenesis and wound healing. Recent studies shed light on the pharmacodynamics of histatin-1, by identifying the endothelial receptor that it binds and downstream signaling. This perspective will discuss current evidence on the role of histatins in wound healing and angiogenesis, emphasizing their impact on regenerative medicine.

## Introduction

Histatins are antimicrobial peptides, naturally encountered in human saliva, which depict a variety of functions including antifungal activity, maintenance of dental enamel homeostasis, and wound healing enhancement.^1^ Compelling evidence in epithelial cell models has shown that a subgroup of histatins promotes epithelial cell adhesiveness, re-epithelialization as well as mucosal and skin wound healing *in vitro* and *in vivo* (reviewed in the studies by Pan L. et al,^2^ Torres P et al.,^3^ and van Dijk I.A. et al.^4^). Interestingly, more recent studies show that one of the most abundant histatins in saliva, histatin-1, harbors potent proangiogenic activity, which is similar to that elicited by the prototypal angiogenic factor, vascular endothelial growth factor A (VEGF-A) and that this is mainly attributed to its migration-promoting effects in endothelial cells.^5^^,^^6^ By promoting endothelial cell adhesion, spreading, and migration, histatin-1 induces vascular tube formation *in vitro* and angiogenesis *in vivo*,^5^^,^^6^ events that are associated with the activation of conserved signaling pathways in endothelial cells.^6^ The variety of effects that histatin-1 triggers in endothelial cells, mostly related to the angiogenic responses, open new opportunities to the field of regenerative medicine. Supported on the basis that angiogenesis is a crucial step in soft and hard tissue repair, several studies have shown that histatin-1 promotes skin wound healing in rat and mice models of diabetes^7^^,^^8^ and rodent burn models,^9^ and that it promotes bone repair *in vivo*,^10^^,^^11^ where its administration is associated with increased vascularization *in vivo*. The increasing number of studies that highlight the potential uses of histatin-1 in tissue repair, along with the plethora of patent applications claiming for therapeutic uses of histatin-1 in regenerative medicine, raises an important concern regarding the appropriate use of this molecule. This is critical because, unlike the mode of action, little is known about the molecular mechanism by which this molecule promotes angiogenesis. To this end, it will be relevant to understand the pharmacodynamics of histatin-1 and the cognate receptor(s) in endothelial cells, in order to get a rational design of therapies based on this peptide.

This perspective summarizes past and current literature regarding the relevance of histatin-1 and related histatins in the wound healing process, with emphasis on their recently acknowledged proangiogenic effects and their impact in the field of regenerative medicine.

## Saliva: A source of factors that contribute to maintain mucosal homeostasis

**Figure 1:**
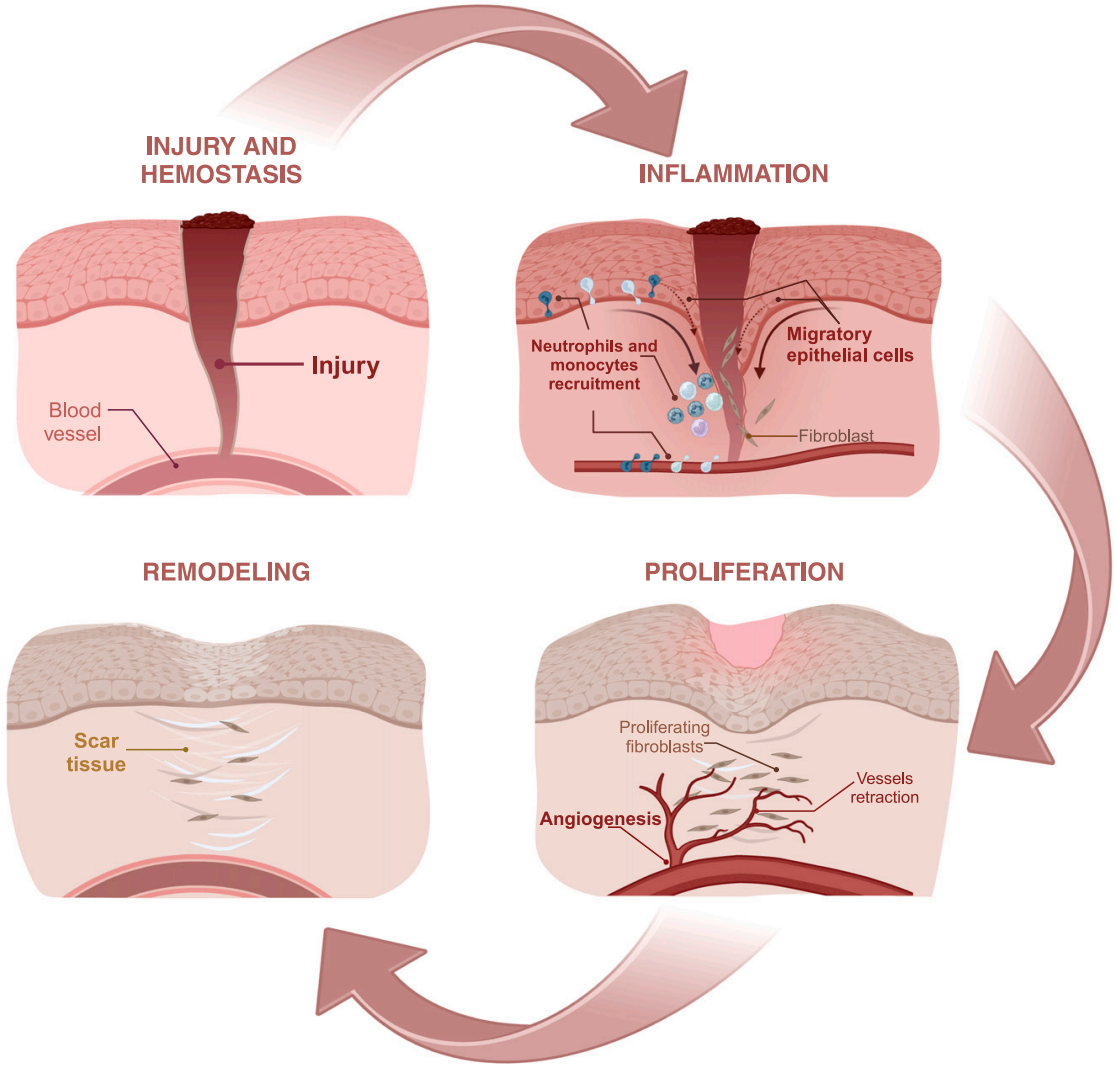
Wound healing The different phases of epithelial wound healing are described. These include hemostasis, inflammation, proliferation, and remodeling (see the main text for details).

Wounds in the oral mucosa heal faster and more efficiently than those in other epithelial tissues such as the skin.^12^^,^^13^^,^^14^ This is interesting, because regardless of the tissue, the wound-healing process proceeds via the same spatiotemporally regulated phases, which include hemostasis, inflammation, proliferation, and remodeling (Figure 1). Thus, the differences in the wound-healing capacities between oral mucosa and other tissues are mainly due to both intrinsic factors, including tissue architecture, and extrinsic factors such as the presence of saliva.^12^^,^^14^ Saliva is critical to the maintenance of oral mucosal homeostasis, as it plays a variety of roles beyond the canonical effects in mechanical lubrication, oral hygiene, chewing function, digestion, and buffering capacity, but it is also the source of antimicrobial and wound healing-promoting factors, which are relevant to the maintenance of oral mucosal function.^12^^,^^15^ Indeed, popular cultures have implemented “therapeutic uses” of saliva, derived from either human or domestic animals, for the treatment of wounds.^16^ Besides the popular belief that saliva is a “wound healing fluid”, compelling evidence supports the notion that saliva promotes epithelial wound healing, based on studies obtained using *in vitro* and *in vivo* models.^12^^,^^17^^,^^18^ Saliva contains a plethora of molecules, including growth factors and antimicrobial peptides, which contribute to the improved wound-healing effect.^12^^,^^13^^,^^14^^,^^15^ These molecules have impacts at the different stages of wound repair. For example, the presence of tissue factor, a known initiator of the coagulation cascade, gives saliva the hemostatic potential necessary for wound repair.^19^^,^^20^ Regarding antimicrobial molecules, multiple reports refer to the presence of these peptides and their positive impact on the maintenance of oral mucosal homeostasis (for further information see the reviews by Vila T. et al.^15^ and Dawes C. et al.^21^). Moreover, human saliva contains a great variety of peptides and proteins that favor cell migration and proliferation, as central processes to the proliferative phase of wound repair.^14^^,^^15^^,^^22^ Among these molecules, it is well known the role of growth factors, including epithelial growth factor (EGF) and VEGF,^12^^,^^17^ in conjunction with cathelicidin (LL-37)^23^ and the trefoil factor-3.^24^ However, within the group of salivary molecules that contribute to the maintenance of oral tissues, histatins are particularly remarkable, as these peptides have been attributed with a plethora of functions in oral homeostasis and several effects in different cell types (reviewed in^1^^,^^3^^,^^15^).

## Histatins, salivary peptides with a variety of biological effects

**Figure 2:**
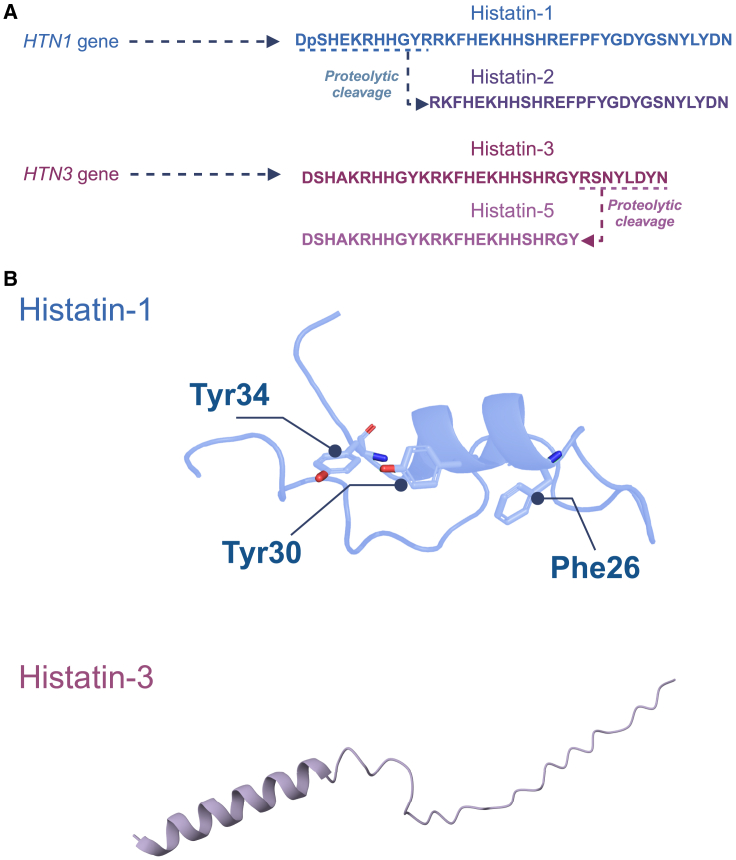
Human histatins (A) Amino acid sequence of main human histatins. The two histatin genes, HTN1 and HTN3, are indicated with their protein products, histatin-1 and histatin-3, respectively. Derivative fragments of these histatins (histatin-2 and histatin-5) are also shown. (B) The 3D structure of histatin-1 is shown, highlighting residues Phe26, Tyr30, and Y34, which are critical for biological activity in endothelial cells. The 3D model was previously proposed by Mateluna et al., in molecular modeling studies.^5^ The 3D structure of full-length histatin-3 is shown and it was obtained from AlphaFold Protein Structure Database (repository code: AF_AFP15516F1).^29^^,^^30^

Histatins are a family of histidine-rich peptides, mainly expressed in human saliva and to a lesser extent, in other body fluids, such as tears^25^^,^^26^^,^^27^^,^^28^ (reviewed in the studies by Melino S. et al.^1^ and Torres P. et al.^3^). From within this family of peptides, histatin-1 and histatin-3 are protein products encoded by the genes HTN1 and HTN3, respectively, whereas proteolytic cleavage of these peptides yields smaller fragments, including histatin-2 (derived from histatin-1) and histatins -4, -5, -6, -7, -8, -9, -10, -11, and -12 (derived from histatin-3)^1^. The sequences of main histatins, including histatin-1, -2, -3, and -5, are shown in Figure 2.

These peptides have been extensively studied for their roles in the innate immune system, particularly for their antimicrobial properties and for their capability to bind copper(II) and zinc(II) metals. Structure and functional analyses based on histatin-5 indicate that these peptides contain two relevant metal-binding motifs, which are located within the NH2-terminal region and that include a copper(II)/nickel(II) binding motif (D-X-H) and a zinc(II) binding motif (HEXXH).^1^ Histatin-5 and histatin-3 are known for their potent antifungal activity against various pathogens, including *Candida albicans*, *Candida krusei*, *Candida glabrata*, *Cryptococcus neoformans*, and *Aspergillus fumigatus*.^26^^,^^31^^,^^32^ The effectiveness of histatin-5 is highlighted by its correlation with the prevalence of *Candida albicans* in HIV-positive^33^ and COVID-19-positive patients,^34^ which showed reduced levels of this histatin along with an increased incidence of fungal infection. In addition to the antifungal properties, histatin-5 exhibits significant antibacterial activity against *Enterococcus faecium*, *Enterobacter cloacae*, *Pseudomonas aeruginosa*, *Acinetobacter baumannii*, and *Staphylococcus aureus*.^35^^,^^36^ Histatin-5 also inhibits proteases from *Porphyromonas gingivalis* (Arg-gingipain and Lys-gingipain) and *Clostridium histolyticum* (clostripain).^37^ These findings underscore the importance of histatins as crucial antimicrobial peptides in the oral cavity.

Another archetypal histatin, histatin-1, is primarily recognized for its various roles in dental enamel homeostasis and antimicrobial activity (reviewed in the study by Melino S. et al.^1^). On the one hand, histatin-1 contains a phosphorylated serine residue (Ser-2) that facilitates its binding to calcium hydroxyapatite,^26^^,^^38^ which allows histatin-1 to integrate into the acquired enamel pellicle and that plays a vital role in the protection of dental enamel.^39^^,^^40^ The salivary pellicle provides buffering capacity and acts as a reservoir for calcium ions, aiding in the remineralization of incipient carious lesions.^40^ Indeed, it has been shown that patients at high risk of caries have lower concentrations of histatin-1, as compared with those without caries,^41^ and histatin-1 levels increase in whole saliva following treatment for early childhood caries.^42^

Furthermore, histatin-1 expression throughout an individual’s life is associated with the dental eruption process.^43^ Histatin-1 levels start to increase around 7 months of age and peak between the first and sixth years of life.^43^ This timing aligns with the eruption of deciduous teeth in the first year and the eruption and replacement of permanent teeth around 6 years.^43^^,^^44^ Initially, it seems that the relationship between histatin-1 and tooth eruption is primarily due to its role in enamel homeostasis; however, recent studies indicate that during the first year of life, most histatin-1 in saliva is in its non-phosphorylated form.^43^ This is intriguing, because it is currently known that the non-phosphorylated form of histatin-1 is capable of inducing cell migration, as it will be described in the following text,^45^^,^^46^ and this is a relevant aspect of wound repair, which might affect the onset of deciduous tooth eruption.^43^^,^^45^

As aforementioned, histatins have been historically studied in the context of their antimicrobial properties and because of their role in the maintenance of enamel homeostasis. However, evidence accumulated in the last decade has shown that some histatins are inducers of epithelial wound healing via stimulation of a common mechanism in tissue repair: induction of cell migration (reviewed in the studies by Pan L. et al,^2^ Torres P. et al.,^3^ and van Dijk I.A. et al.^47^; Table 1).

**Table 1:** Histatins and their effects in cell and tissue wound healing

Histatin Type	Cell Type	Receptor	Signaling Pathway	Cellular Effects	Reference
**Histatin-1**	Endothelial cells	Vascular Endothelial Growth Factor Receptor 2, (VEGFR-2). Direct binding and involvement of this receptor in histatin-1-dependent effects.	Involvement of the Rin2/Rab5/Rac1 axis and activation of ERK1/2. Nuclear translocation of Nrf2, and decreased interaction of the IP3R1/GRP75/VDAC1 complex.	Cell adhesion, spreading and migration; angiogenesis *in vitro* and *in vivo.* Anti-senescence and antioxidant effects.	Torres et al.^6^, Cao et al.^7^, Zhu et al.^8^, van Dijk et al.^48^, Lin et al.^49^, and Xian et al.^50^
–	Epithelial cells	Involvement of G protein-coupled receptor (GPCR), in conjunction with Sigma-2 Receptor (S2R). Co-regulation of these receptors is suggested.	Activation of ERK1/2 and E-Cadherin has been described. In addition, an anti-apoptotic response (IGF-1/BCL-2/BAX) was observed.	Cell-matrix and cell-cell adhesion, as well as cell migration and viability. Re-epithelialization *in vitro* and *in vivo.*	Zheng et al.^9^, Oudhoff et al.^45^, van Dijk et al.^47^, Shah et al.^51^, Lei et al.^52^, Oydanich et al.^53^, Liu et al.^54^, van Dijk et al.^55^, Pan et al.^56^, Ma et al.^57^, Son et al.^58^, Huang et al.^59^, and Lei et al.^60^
–	Osteoblastic lineage cells	ND	Activation of β-catenin signaling. In addition, cytoprotective roles have been associated with decreased caspase-3 activation.	Cell adhesion and migration. Induction of osteoblastic differentiation markers, including ALP, Runx2, Osteopontin, Osteocalcin, along with mineralizing phenotype. Bone repair *in vivo*.	Sun et al.^10^, Torres et al.^11^, Castro et al.^61^, Torres et al.^62^, Sun et al.^63^, Sun et al.^64^, van Dijk et al.^55^, Siwakul et al.^65^, Shi et al.^66^, and Du et al.^67^
–	Fibroblasts	ND	Activation of mTOR signaling pathway.	Fibroblast migration and expression of myofibroblast markers *in vitro*.	van Dijk et al.^47^, Arab et al.^68^, Lin et al.^49^, and Cheng et al.^69^
–	Macrophages	ND	Decreased phosphorylation of JNK and p38; negative regulation of NF-kB.	Decreased pro-inflammatory markers; macrophage transition from M1 to M2.	Wu et al.^70^ and Lee et al.^71^
–	Mesenchymal stem cells	ND	Involvement of p38 and ERK1/2 is suggested.	Cell adhesion.	Wang et al.^72^
**Histatin-2**	Fibroblasts	ND	–	Cell migration.	Oudhoff et al.^23^, Oudhoff et al.^46^, and van Dijk et al.^47^
–	Epithelial cells	GPCR is suggested on the basis of inhibition experiments.	ERK1/2 activation.	Cell adhesion and migration. Re-epithelialization *in vitro.*	Oudhoff et al.^45^, Oudhoff et al.^46^, van Dijk et al.^47^, and Oudhoff et al.^73^
–	Mesenchymal stem cells	ND	–	Cell migration.	Boink et al.^74^
**Histatin-3**	Fibroblasts	ND	Histatin-3/HSC70 complex binds to p27, and is degraded, allowing the cell cycle to continue (G_1_/S transition).	Cell proliferation.	Imamura et al.^75^
**Histatin-5**	Chondrocytes	ND	–	Cell proliferation; synergic effects with EGF.	Murakami et al.^76^
–	Epithelial cells	ND	ERK1/2 activation.	Cell adhesion and migration. Re-epithelialization *in vitro.*	Shah et al.^77^

N.D., non-determined.

## Histatins, wound healing and cell migration

**Figure 3:**
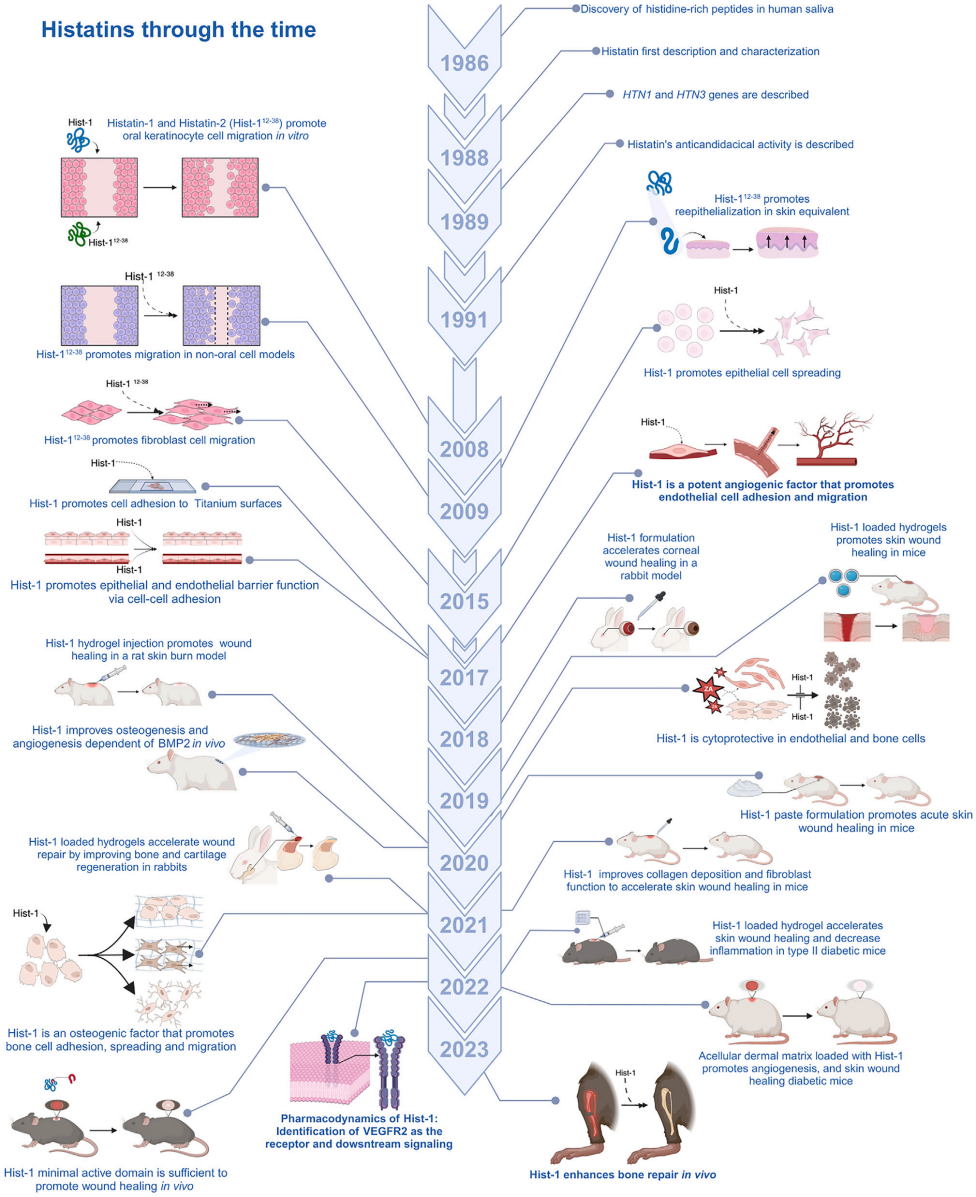
Timeline: retrospective analysis of histatin-dependent effects in tissue repair The timeline shows the main findings related to the roles histatins in cell and tissue repair. These include the effects at the cellular level, namely cell adhesion, spreading, and migration. Also, the effects of histatins in soft and mineralized tissue repair are shown, along with the different therapeutic strategies that have been proposed in animal models. The impact of histatins on angiogenesis and the consequences in tissue repair are highlighted.

The role of histatins in cell migration and wound healing was first documented by Oudhoff and colleagues in 2008, as these peptides were shown as the major factors present in human saliva that contribute to wound healing *in vitro*.^45^ This study and a subsequent report showed that histatin-1 and its derivative fragment, histatin-2, promote cell migration in a wound-scratch assay in oral keratinocytes and increase re-epithelialization in a skin equivalent model.^45^^,^^73^ Subsequent studies extended the migration-promoting activity of histatin-1 and the derived 12–38 residue fragment (histatin-2) to other oral cells and non-oral cells, including oral and dermal keratinocytes, gingival and skin fibroblasts,^23^^,^^45^^,^^46^^,^^73^ bone and primary mesenchymal cells,^61^^,^^62^ corneal epithelial cells,^51^ human periodontal ligament cells,^68^ as well as endothelial cells^6^ (a timeline of histatin-associated effects in cell migration and wound healing is provided in Figure 3, and a summary of the literature is shown in Table 1). This body of evidence led to the definition of histatin-1 as a general promoter of cell migration (reviewed in the study by Torres P. et al.^3^). Moreover, histatin-1 and some derivative fragments have been shown to increase cell adhesion and spreading both in the absence and presence of extracellular matrix, as shown in epithelial, bone lineage, and endothelial cells,^6^^,^^47^^,^^62^ whereas histatin-1 itself was shown to promote cell-cell adhesion via tight and adherens junctions in epithelial cells.^48^

The extent of the effects that histatin-1 triggers in cells other than those located in oral tissues provide new opportunities to explore the potential uses of this peptide in regenerative medicine. In the following paragraphs, examples of proposed therapeutical uses for histatin-1 in different models are provided. Importantly, most evidence points toward the effects of this molecule in the re-epithelialization phase of wound healing with emphasis on the angiogenic responses.

### Histatins in soft tissue repair

Pioneering studies in a full-skin wound model based on an equivalent that resembles human skin indicated that the 12–38 amino acid fragment derived from histatin-1 promotes reepithelialization in skin keratinocytes.^73^ Subsequent studies in animal models including mice, rats, and rabbits supported the notion that histatin-1 and related peptides promote soft tissue repair in general, as it will be described in the next paragraphs. In mice, treatment of full-thickness skin wounds with histatin-1, administered via conjugation in thermosensitive thiolated chitosan hydrogels, enhanced wound healing by increasing blood vessel formation (CD31 and VEGFA-positive staining) and collagen fiber alignment.^49^ Also, by using the same model of full-thickness skin wound healing in C57/BL6 mice, the topic application of a solution containing 10 μM histatin-1, promoted wound healing and improved the mechanical properties of healed skin, by increasing collagen deposition, granulation tissue, and fibroblast number within the wounded area.^52^^,^^69^ These observations were in agreement with the observation that histatin-1 promotes fibroblast differentiation and contractility in collagen gels,^69^ and they are also in agreement with early *in vitro* studies showing that histatin-1 harbors motogenic activity (i.e., migration-promoting effects) in dermal and gingival fibroblasts.^46^^,^^74^

The application of histatin-1-functionalized materials has been explored by using an acellular dermal matrix, as a drug delivery carrier of histatin-1, in a model of wound healing in diabetic Sprague-Dawley rats, which showed increased angiogenesis and minimal scarring width *in vivo*.^7^ Specifically, functionalization of an acellular dermal matrix with histatin-1 fused to a collagen-binding domain (for increased affinity toward the collagen-rich acellular dermal matrix), permitted sustained release of histatin-1, vascular endothelial cell adhesion and migration, extracellular collagen accumulation, and endothelial tube formation *in vitro*.^7^ These observations are reminiscent of those obtained in C57 mice, where the administration of histatin-1, embedded in an acellular dermal matrix, increased the wound healing capacity in full-thickness wounds, though not to the same extent as the effects observed with topic administration of soluble histatin-1 (10 μM), which showed the best proangiogenic and anti-inflammatory responses.^52^ These discrepancies might be due to the lack of a sustained-release mechanism of histatin-1.

Alternatively, the role of histatin-1 in preclinical rodent models of wound healing has been explored in the context of diabetes and acute burning. On the one hand, gelatin/chitosan-based hydrogels loaded with histatin-1 were shown to accelerate full-thickness wound healing in db/db congenital diabetic mice, events that were associated with downregulation of pro-inflammatory mediators and upregulation of CD31 and α-SMA (α-smooth muscle actin), which suggests a potential application of this molecule in diabetic wounds.^8^ On the other hand, in an established model of rat burn wound healing, treatment with a host-guest gelatin hydrogel loaded with histatin-1 promoted wound healing by inhibiting the expression of pro-inflammatory factors and by increasing the vascularization at skin burn wound sites.^9^

The effects of histatins in soft tissue repair have been extended to models beyond the skin tissue, such as corneal epithelium, where soluble histatin-1 was found to promote corneal epithelium wound healing in a rabbit model.^53^ These observations are in agreement with previous findings *in vitro*, where histatin-1 promoted corneal epithelial cell spreading and migration in a wound-scratch assay^51^ and it calls attention, based on earlier evidence reporting the expression of this molecule in human lacrimal epithelium.^27^ Intriguingly, this wound-healing capacity in corneal epithelial cells was also extended to another histatin, histatin-5, as it was found to promote corneal wound healing *in vitro* and in C57BL/6 mice.^77^ The effects of histatins in other epithelial tissues have been extended to the context of peri-implantitis, where histatin-1, embedded in a small intestine mucosa hydrogel, favored epithelial sealing around implant surfaces, thereby decreasing peri-implantitis.^54^ Whether the wound-healing capacities of histatins are extended to other soft tissues remains to be explored and will require future research.

### Histatins in mineralized tissue repair

Besides the widely documented effects of histatins in soft tissue repair, accumulating evidence points toward the therapeutical application of these molecules in hard tissue repair. Studies in major bones, cartilage, and subchondral bone, as well as in dental tissues are becoming increasingly reported, as follows. Early studies showed that histatin-1 and derivative peptides promote bone-lineage cell adhesion and spreading onto different surfaces, including glass and bioinert surfaces,^55^^,^^63^^,^^64^ non-treated and sandblasted-acid etched titanium surfaces,^55^^,^^63^ and extracellular matrix-coated surfaces.^62^ In fact, immobilized peptides derived from histatin-1, attached to titanium surfaces promote cell spreading and expression of osteogenic genes in murine preosteoblasts.^65^ These observations become relevant in the field of dental implantology and osseointegration, since histatin-1-derivative peptides have been shown to promote trabecular bone formation in a canine model of titanium-based implants.^78^ In this context, histatin-1 was recently reported as a novel osteogenic molecule that promotes bone cell adhesion and migration, as well as the expression of genes involved in osteogenic differentiation, as shown in MC3T-E1 murine preosteoblasts and SAOS-2 human osteosarcoma cells.^62^ Moreover, this peptide promoted mineralization *in vitro*, as shown in osteoblastic-lineage cells and primary mesenchymal cells derived from the dental pulp and apical papilla.^62^ These findings were further validated at the preclinical level, where histatin-1 accelerated bone regeneration in a murine model of non-critical size defect.^11^ Alternatively, studies in rats showed that co-administration of histatin-1 improved the extent of osteogenesis induced by the osteogenic agent BMP2 (bone morphogenetic protein 2), as shown in a model of ectopic bone formation.^10^ Interestingly, regardless of the model (ectopic or orthotopic bone formation *in vivo*), the observations were associated with improved blood vessel formation, as it will be mentioned in the following text.^10^^,^^11^ In summary, current evidence points toward the notion that histatin-1 and derived peptides behave as novel osteoinductive factors at both the *in vitro* and *in vivo* levels.

The effects of histatins in mineralized tissue repair have been extended to other tissues including cartilage and subchondral bone in temporomandibular joints, as the administration of histatin-1, functionalized in a methacrylate gelatin hydrogel promoted bone and cartilage regeneration in critical-size osteochondral defects in temporomandibular joints of New Zealand white rabbits.^66^ This study showed that the effects of histatin-1 are dose dependent, in a manner that differentially impacted either bone or cartilage regeneration, an issue that is intriguing based on the acknowledged relevance of vascular formation during either process.^79^ On the other hand, in a rat model of osteoarthritis, histatin-1 attenuated both cartilage and bone deconstruction via decreased macrophage infiltration.^70^ These findings are in agreement with the anti-inflammatory effects of histatin-1, as this peptide has been found to promote the M1 to M2 transition in macrophages, and it was also shown to decrease the secretion of pro-inflammatory cytokines.^71^

Taken together, the evidence provided at both the *in vitro* and *in vivo* levels suggests that histatin-1 is a general promoter of wound healing in mineralized and soft tissues. Remarkably, most preclinical studies coincide in that the effects of histatin-1 in these tissues are attributed to histatin’s proangiogenic activity, which is relevant, because angiogenesis is a critical process required during wound healing. Most of this evidence is supported by seminal studies showing that histatin-1 is a proangiogenic factor.^6^

## Histatin-1, a novel proangiogenic factor

Compelling evidence at both *in vitro* and *in vivo* levels supports the notion that a subgroup of histatins, namely histatin-1 and to a lesser extent histatin-2, are proangiogenic, as it will be described in the following text. Pioneering studies in 2017 showed for the first time that histatin-1 is a potent angiogenic factor, with effects that are reminiscent of those elicited by the prototypal angiogenic factor, VEGFA.^6^ Specifically, histatin-1 stimulates cellular and molecular events that contribute to angiogenesis, including endothelial cell adhesion, spreading, and migration, as well as endothelial tube morphogenesis *in vitro* and angiogenesis *in vivo*.^6^ In doing so, salivary concentrations of histatin-1 (5–10 μM) accelerated the kinetics of endothelial cell adhesion and spreading on fibronectin-coated surfaces, which was paralleled by increased endothelial cell migration and the activation of a conserved signaling cascade that stems at the endosomal compartments (the cell signaling induced by histatins will be discussed in the following section). These observations were confirmed in different cell models, including the endothelial cell line EA.hy926 and human umbilical vein endothelial cells (HUVECs), as well as by using histatin-1 obtained from different sources, including chemical synthesis and the peptide proceeding from healthy donors of human saliva.^6^ Moreover, histatin-1 augmented both endothelial tube formation *in vitro* and angiogenesis in the chick chorioallantoic membrane assay, which was particularly intriguing, because in both cases, the magnitude of the effects triggered by histatin-1 was similar to that observed with VEGFA.^6^ Interestingly, another study published in the same year showed that histatin-1 promotes endothelial cell adhesion, thereby improving endothelial barrier function.^48^ Subsequent studies showed that histatin-1 restores endothelial cell migration and tube formation capacity following exposure to cytotoxic drugs,^61^ which extends the cytoprotective effects of this peptide observed in other non-endothelial cell models.^56^ Moreover, the effects of histatins on endothelial cells have been extended to another related histatin, namely histatin-2 (the derivative fragment of histatin-1), which was capable of inducing endothelial cell migration *in vitro.*^5^

The aforementioned studies constituted the basis for subsequent reports that supported their observations of histatin-associated wound healing capacity on the peptide’s proangiogenic effects *in vivo* (described in the previous section). Specifically, in Sprague-Dawley rats, histatin-1 promoted full-thickness wound healing associated with increased expression of blood vessel markers CD31 and VEGFA.^9^^,^^49^ Similarly, in a C57 mice model of full-thickness wound healing, histatin-1 promoted formation of CD31 and VEGFA-positive blood vessels.^52^ The latter was further extended to a congenital model of diabetic mice and rats, whereby histatin-1 promoted full-thickness wound healing associated with increased vascularization and protein levels of CD31 ^7^^,^^8^. Similar observations have been made in hard tissue, where histatin-1 allowed bone regeneration via increased blood vessel formation^11^ and contributed to BMP-2-induced osteogenesis *in vivo* via increasing VEGFA and CD31 positivity.^10^ Taken together, the evidence supports the notion that histatin-1 promotes wound healing in soft and mineralized tissue via induction of blood vessel formation.

The current view that histatin-1 improves wound healing (endogenously in the oral mucosa or upon therapeutic administration in other tissues) via promoting angiogenesis and endothelial cell responses is referred to as the ***modeof action***. Nevertheless, the mechanisms by which histatin-1 promotes endothelial cell migration and angiogenesis, i.e., the ***mechanism of action***, remain poorly understood. In this context, recent studies by our group identified the receptor for histatin-1 in endothelial cells, as the vascular endothelial growth factor receptor 2 (VEGFR2).^5^ By using pharmacological inhibition, knockdown, and direct binding approaches, VEGFR2 was shown as the cognate receptor for histatin-1 and to be required for histatin-1-dependent endothelial cell signaling, migration, and angiogenesis *in vitro*.^5^ These findings provide relevant insights into the molecular pharmacology of histatin-1, representing a first approach to design, modify, and improve structural elements contained in this molecule, to get better outcomes for pharmacological uses.

## Pharmacodynamics of histatin-1 in endothelial cells

The identification of VEGFR2 as the histatin-1 receptor in endothelial cells paved new roads to the pharmacodynamic studies of these molecules in angiogenesis, as a common denominator in tissue regeneration. Since VEGFR2 plays a pivotal role in regulating angiogenesis, the binding and activation of this receptor by histatin-1 offer new possibilities for therapeutic interventions requiring the improvement of new blood vessel formation.

**Figure 4:**
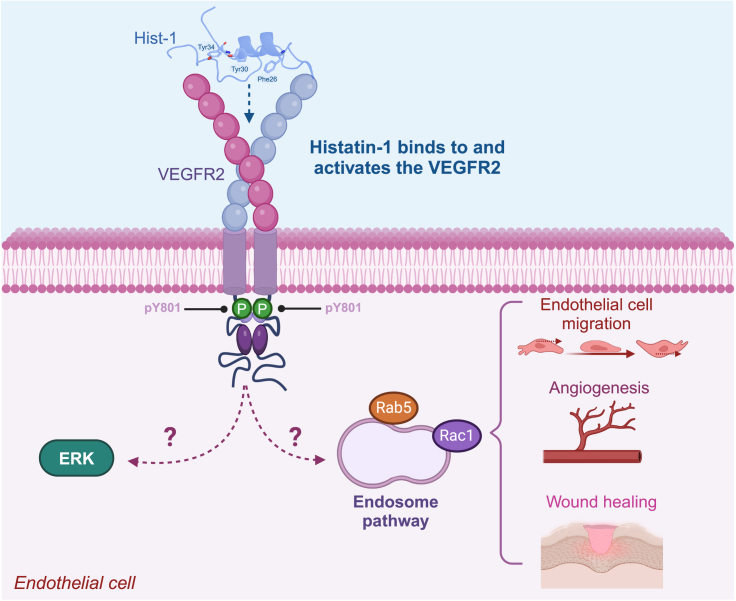
Pharmacodynamics of histatin-1 The image summarizes the findings that histatin-1 is a potent angiogenic factor, which binds to the VEGFR2, leading to the activation of downstream signaling. While ERK and Rab5/Rac-dependent signaling have been described downstream histatin-1, their sequential activation and requirement of VEGFR2 remain to be explored.

Structure analysis of histatin-1 revealed that the binding to VEGFR2 occurs via three aromatic residues that are analogic to those described in the endogenous ligand VEGFA (Figures 2B and 4). Specifically, residues Phe26, Tyr30, and Tyr34 are essential for histatin-1-dependent effects in endothelial cells, including migration and angiogenesis *in vitro*, as shown by mutational analyses.^5^ These observations are intriguing, because it has been demonstrated that the binding and activation of VEGFR2 lead to a plethora of downstream phosphorylation events on the receptor’s cytoplasmic tail, which instead trigger differential signaling pathways and endothelial cell responses, ranging from proliferation and viability, through migration and vascular morphogenesis.^80^ The observation that histatin-1 rather promotes a subset of effects in endothelial cells, including adhesion, spreading, and migration, but not proliferation, is intriguing, because it provides insights into the phosphorylation events that may account within the receptor.^5^^,^^6^ To date, only one tyrosine phosphorylation has been reported in VEGFR2, namely Tyr801^5^, and hence, further research is required to identify other possible targets of histatin-1. As mentioned, the different phosphorylatable tyrosine residues in VEGFR2 are involved in the differential activation of downstream signaling pathways. In this respect, it is interesting that histatin-1 activates two signaling branches, which include ERK1/2 on the one hand, and endosomal signaling by Rab5/Rac on the other hand^6^ (Figure 4). Particularly, activation of the Rab5/Rac signaling axis is required for histatin-1 to induce endothelial cell migration.^6^ However, whether these and other yet-to-be-identified signaling pathways are placed downstream binding of VEGFR2, remains to be determined. Interestingly, in non-endothelial cells, histatin-1 was shown to activate components of the MAPK pathway, an event associated with cell migration.^45^^,^^57^^,^^63^^,^^73^

Intriguingly, unlike endothelial cells, in epithelial cells, histatin-1 has been proposed to act via a G-coupled protein receptor (GCPR), as shown in inhibition experiments with pertussis toxin.^57^^,^^73^ However, these data should be carefully interpreted, since direct binding of histatin-1 to GCPR has not been demonstrated yet, but only the dependency of this receptor. In the same model of epithelial cells, histatin-1 and histatin-2 were shown to be targeted to subcellular compartments, including the endoplasmic reticulum and mitochondria,^81^ and this targeting has been associated with the capacity of these peptides to induce cellular metabolic activity in a GCPR-dependent manner.^57^^,^^81^ Accordingly, in epithelial cells, histatin-1 binds to the sigma 2 receptor (TMEM97) at the endoplasmic reticulum, and this binding is required for epithelial cell migration.^58^ Whether similar mechanisms operate in endothelial cells remains to be determined, along with the functional requirement of the receptor.

As aforementioned, former studies on the pharmacodynamics of histatin-1 in endothelial cells reveal a sophisticated mechanism involving the activation of VEGFR2 and multiple downstream signaling pathways.^5^^,^^6^ Based on its histidine-rich sequence, histatin-1 might interact with negatively charged surfaces at the plasma membrane, thereby enabling anchoring and subsequent activation of VEGFR2, allowing the peptide to be further endocytosed. These possibilities need to be further assessed, as they would allow us to understand the stability and efficacy of this peptide for future therapeutic applications. This structural feature is not only vital for its function in angiogenesis, but also provides a foundation for innovative therapeutic applications, such as the design of mimetic peptides that can mimic or potentiate the effects of histatin-1 in tissues where angiogenesis is compromised.

## Future directions and perspectives

Increasing evidence raises the view that the salivary peptide histatin-1 is a novel factor that promotes wound healing in soft and hard tissues by inducing a variety of cell responses in different cell types. The findings that histatin-1 increases re-epithelialization in epithelial cells, osteogenic differentiation in bone-lineage cells, and angiogenesis via endothelial cell responses, highlighted this protein as a multifunctional molecule with therapeutic uses in regenerative medicine. Of note, emphasis should be made on the appropriate design and formulation of histatin-1-based therapies, as the pharmacology of this fascinating molecule is beginning to be elucidated.

Besides the applicability of histatin-1 as a therapeutic agent to improve wound healing, the identification of this peptide as a proangiogenic factor provides insights that help to understand the basis of efficient wound healing in the oral mucosa, as this tissue is known to heal faster and more efficiently than skin.^14^ In this respect, it remains intriguing whether histatin-1-driven angiogenesis accounts for better wound healing in the oral mucosa, since coordinated and time-restricted, rather than exacerbated and continuous angiogenesis, drives efficient and scar-free wound healing.^82^ Hence, future studies will be required to understand whether histatin-1 acts as a spatiotemporal regulator of angiogenesis in a clinical context and whether this event is responsible for improved wound healing in the oral mucosa. Finally, the identification and molecular characterization of histatin-1’s angiogenic activity open new avenues for both, to understand the basic biology of wound healing and to provide new opportunities to design novel therapeutic approaches in regenerative medicine.

## Acknowledgments

This work was supported by the 10.13039/501100002850National Fund for Scientific and Technological Development (FONDECYT) 1220517; the Advanced Center for Chronic Diseases*,* FONDAP-ACCDiS 15130011; 10.13039/100019783National Agency for Research and Development (ANID)- Millennium Science Initiative Program - ICN09_016/ICN 2021_045 "10.13039/501100013928Millennium Institute on Immunology and Immunotherapy".

## Author contributions

H.T., P.T., C.M., M.C, and V.T wrote and reviewed the manuscript.

## Declaration of interests

The authors declare no competing interest.

## References

[1] Melino S., Santone C., Di Nardo P., Sarkar B. (2014). Histatins: salivary peptides with copper(II)- and zinc(II)-binding motifs: perspectives for biomedical applications. FEBS J..

[2] Pan L., Zhang X., Gao Q. (2021). Effects and mechanisms of histatins as novel skin wound-healing agents. J. Tissue Viability.

[3] Torres P., Castro M., Reyes M., Torres V.A. (2018). Histatins, wound healing, and cell migration. Oral Dis..

[4] van Dijk I.A., Veerman E.C.I., Reits E.A.J., Bolscher J.G.M., Stap J. (2018). Salivary peptide histatin 1 mediated cell adhesion: a possible role in mesenchymal-epithelial transition and in pathologies. Biol. Chem..

[5] Mateluna C., Torres P., Rodriguez-Peña M., Silva P., Matthies D.J., Criollo A., Bikker F.J., Bolscher J.G.M., Wilson C.A.M., Zapata-Torres G., Torres V.A. (2022). Identification of VEGFR2 as the Histatin-1 receptor in endothelial cells. Biochem. Pharmacol..

[6] Torres P., Díaz J., Arce M., Silva P., Mendoza P., Lois P., Molina-Berríos A., Owen G.I., Palma V., Torres V.A. (2017). The salivary peptide histatin-1 promotes endothelial cell adhesion, migration, and angiogenesis. Faseb. J..

[7] Cao Y., Shi X., Zhao X., Chen B., Li X., Li Y., Chen Y., Chen C., Lu H., Liu J. (2022). Acellular dermal matrix decorated with collagen-affinity peptide accelerate diabetic wound healing through sustained releasing Histatin-1 mediated promotion of angiogenesis. Int. J. Pharm..

[8] Zhu S., Yu C., Zhao M., Liu N., Chen Z., Liu J., Li G., Deng Y., Sai X., Huang H. (2022). Histatin-1 loaded multifunctional, adhesive and conductive biomolecular hydrogel to treat diabetic wound. Int. J. Biol. Macromol..

[9] Zheng Y., Yuan W., Liu H., Huang S., Bian L., Guo R. (2020). Injectable supramolecular gelatin hydrogel loading of resveratrol and histatin-1 for burn wound therapy. Biomater. Sci..

[10] Sun P., Shi A., Shen C., Liu Y., Wu G., Feng J. (2020). Human salivary histatin-1 (Hst1) promotes bone morphogenetic protein 2 (BMP2)-induced osteogenesis and angiogenesis. FEBS Open Bio.

[11] Torres P., Flores V., Flores T., Silva P., González L., Córdova L.A., Reyes M., Torres V.A. (2023). The salivary peptide histatin-1 enhances bone repair in vivo. Biochem. Biophys. Res. Commun..

[12] Brand H.S., Ligtenberg A.J.M., Veerman E.C.I. (2014). Saliva and wound healing. Monogr. Oral Sci..

[13] Glim J.E., van Egmond M., Niessen F.B., Everts V., Beelen R.H.J. (2013). Detrimental dermal wound healing: what can we learn from the oral mucosa?. Wound Repair Regen..

[14] Waasdorp M., Krom B.P., Bikker F.J., van Zuijlen P.P.M., Niessen F.B., Gibbs S. (2021). The Bigger Picture: Why Oral Mucosa Heals Better Than Skin. Biomolecules.

[15] Vila T., Rizk A.M., Sultan A.S., Jabra-Rizk M.A. (2019). The power of saliva: Antimicrobial and beyond. PLoS Pathog..

[16] Verrier L. (1970). Dog licks man. Lancet.

[17] Keswani S.G., Balaji S., Le L.D., Leung A., Parvadia J.K., Frischer J., Yamano S., Taichman N., Crombleholme T.M. (2013). Role of salivary vascular endothelial growth factor (VEGF) in palatal mucosal wound healing. Wound Repair Regen..

[18] Rodrigues Neves C., Buskermolen J., Roffel S., Waaijman T., Thon M., Veerman E., Gibbs S. (2019). Human saliva stimulates skin and oral wound healing in vitro. J. Tissue Eng. Regen. Med..

[19] Berckmans R.J., Sturk A., van Tienen L.M., Schaap M.C.L., Nieuwland R. (2011). Cell-derived vesicles exposing coagulant tissue factor in saliva. Blood.

[20] Yu Y., Gool E., Berckmans R.J., Coumans F.A.W., Barendrecht A.D., Maas C., van der Wel N.N., Altevogt P., Sturk A., Nieuwland R. (2018). Extracellular vesicles from human saliva promote hemostasis by delivering coagulant tissue factor to activated platelets. J. Thromb. Haemostasis.

[21] Dawes C., Pedersen A.M.L., Villa A., Ekström J., Proctor G.B., Vissink A., Aframian D., McGowan R., Aliko A., Narayana N. (2015). The functions of human saliva: A review sponsored by the World Workshop on Oral Medicine VI. Arch. Oral Biol..

[22] Guo S., Dipietro L.A. (2010). Factors affecting wound healing. J. Dent. Res..

[23] Oudhoff M.J., Blaauboer M.E., Nazmi K., Scheres N., Bolscher J.G.M., Veerman E.C.I. (2010). The role of salivary histatin and the human cathelicidin LL-37 in wound healing and innate immunity. Biol. Chem..

[24] Storesund T., Hayashi K., Kolltveit K.M., Bryne M., Schenck K. (2008). Salivary trefoil factor 3 enhances migration of oral keratinocytes. Eur. J. Oral Sci..

[25] Kalmodia S., Son K.N., Cao D., Lee B.S., Surenkhuu B., Shah D., Ali M., Balasubramaniam A., Jain S., Aakalu V.K. (2019). Presence of Histatin-1 in Human Tears and Association with Aqueous Deficient Dry Eye Diagnosis: A Preliminary Study. Sci. Rep..

[26] Oppenheim F.G., Xu T., McMillian F.M., Levitz S.M., Diamond R.D., Offner G.D., Troxler R.F. (1988). Histatins, a novel family of histidine-rich proteins in human parotid secretion. Isolation, characterization, primary structure, and fungistatic effects on Candida albicans. J. Biol. Chem..

[27] Shah D., Ali M., Pasha Z., Jaboori A.J., Jassim S.H., Jain S., Aakalu V.K. (2016). Histatin-1 Expression in Human Lacrimal Epithelium. PLoS One.

[28] vanderSpek J.C., Wyandt H.E., Skare J.C., Milunsky A., Oppenheim F.G., Troxler R.F. (1989). Localization of the genes for histatins to human chromosome 4q13 and tissue distribution of the mRNAs. Am. J. Hum. Genet..

[29] Jumper J., Evans R., Pritzel A., Green T., Figurnov M., Ronneberger O., Tunyasuvunakool K., Bates R., Žídek A., Potapenko A. (2021). Highly accurate protein structure prediction with AlphaFold. Nature.

[30] Varadi M., Bertoni D., Magana P., Paramval U., Pidruchna I., Radhakrishnan M., Tsenkov M., Nair S., Mirdita M., Yeo J. (2024). AlphaFold Protein Structure Database in 2024: providing structure coverage for over 214 million protein sequences. Nucleic Acids Res..

[31] Helmerhorst E.J., Reijnders I.M., van't Hof W., Simoons-Smit I., Veerman E.C., Amerongen A.V. (1999). Amphotericin B- and fluconazole-resistant Candida spp., Aspergillus fumigatus, and other newly emerging pathogenic fungi are susceptible to basic antifungal peptides. Antimicrob. Agents Chemother..

[32] Puri S., Edgerton M. (2014). How does it kill?: understanding the candidacidal mechanism of salivary histatin 5. Eukaryot. Cell.

[33] Khan S.A., Fidel P.L., Thunayyan A.A., Varlotta S., Meiller T.F., Jabra-Rizk M.A. (2013). Impaired Histatin-5 Levels and Salivary Antimicrobial Activity against C. albicans in HIV Infected Individuals. J. AIDS Clin. Res..

[34] Alfaifi A.A., Wang T.W., Perez P., Sultan A.S., Meiller T.F., Rock P., Kleiner D.E., Chertow D.S., Hewitt S.M., Gasmi B. (2024). SARS-CoV-2 Infection of Salivary Glands Compromises Oral Antifungal Innate Immunity and Predisposes to Oral Candidiasis. bioRxiv.

[35] Du H., Puri S., McCall A., Norris H.L., Russo T., Edgerton M. (2017). Human Salivary Protein Histatin 5 Has Potent Bactericidal Activity against ESKAPE Pathogens. Front. Cell. Infect. Microbiol..

[36] Welling M.M., Brouwer C.P.J.M., van 't Hof W., Veerman E.C.I., Amerongen A.V.N. (2007). Histatin-derived monomeric and dimeric synthetic peptides show strong bactericidal activity towards multidrug-resistant Staphylococcus aureus in vivo. Antimicrob. Agents Chemother..

[37] Gusman H., Grogan J., Kagan H.M., Troxler R.F., Oppenheim F.G. (2001). Salivary histatin 5 is a potent competitive inhibitor of the cysteine proteinase clostripain. FEBS Lett..

[38] Driscoll J., Zuo Y., Xu T., Choi J.R., Troxler R.F., Oppenheim F.G. (1995). Functional comparison of native and recombinant human salivary histatin 1. J. Dent. Res..

[39] Lee Y.H., Zimmerman J.N., Custodio W., Xiao Y., Basiri T., Hatibovic-Kofman S., Siqueira W.L. (2013). Proteomic evaluation of acquired enamel pellicle during in vivo formation. PLoS One.

[40] Siqueira W.L., Custodio W., McDonald E.E. (2012). New insights into the composition and functions of the acquired enamel pellicle. J. Dent. Res..

[41] Vitorino R., Lobo M.J.C., Duarte J.R., Ferrer-Correia A.J., Domingues P.M., Amado F.M.L. (2005). The role of salivary peptides in dental caries. Biomed. Chromatogr..

[42] Sun X., Huang X., Tan X., Si Y., Wang X., Chen F., Zheng S. (2016). Salivary peptidome profiling for diagnosis of severe early childhood caries. J. Transl. Med..

[43] Messana I., Cabras T., Iavarone F., Manconi B., Huang L., Martelli C., Olianas A., Sanna M.T., Pisano E., Sanna M. (2015). Chrono-proteomics of human saliva: variations of the salivary proteome during human development. J. Proteome Res..

[44] Manconi B., Cabras T., Pisano E., Sanna M.T., Olianas A., Fanos V., Faa G., Nemolato S., Iavarone F., Castagnola M., Messana I. (2013). Modifications of the acidic soluble salivary proteome in human children from birth to the age of 48months investigated by a top-down HPLC-ESI-MS platform. J. Proteonomics.

[45] Oudhoff M.J., Bolscher J.G.M., Nazmi K., Kalay H., van 't Hof W., Amerongen A.V.N., Veerman E.C.I. (2008). Histatins are the major wound-closure stimulating factors in human saliva as identified in a cell culture assay. Faseb. J..

[46] Oudhoff M.J., van den Keijbus P.A.M., Kroeze K.L., Nazmi K., Gibbs S., Bolscher J.G.M., Veerman E.C.I. (2009). Histatins enhance wound closure with oral and non-oral cells. J. Dent. Res..

[47] van Dijk I.A., Nazmi K., Bolscher J.G.M., Veerman E.C.I., Stap J. (2015). Histatin-1, a histidine-rich peptide in human saliva, promotes cell-substrate and cell-cell adhesion. Faseb. J..

[48] van Dijk I.A., Ferrando M.L., van der Wijk A.E., Hoebe R.A., Nazmi K., de Jonge W.J., Krawczyk P.M., Bolscher J.G.M., Veerman E.C.I., Stap J. (2017). Human salivary peptide histatin-1 stimulates epithelial and endothelial cell adhesion and barrier function. Faseb. J..

[49] Lin Z., Li R., Liu Y., Zhao Y., Ao N., Wang J., Li L., Wu G. (2020). Histatin1-modified thiolated chitosan hydrogels enhance wound healing by accelerating cell adhesion, migration and angiogenesis. Carbohydr. Polym..

[50] Xian T., Liu Y., Ye Y., Peng B., Huang J., Liang L., Zhang J., Wu H., Lin Z. (2024). Human Salivary Histatin 1 Regulating IP3R1/GRP75/VDAC1 Mediated Mitochondrial-Associated Endoplasmic Reticulum Membranes (MAMs) Inhibits Cell Senescence For Diabetic Wound Repair. Free Radic. Biol. Med..

[51] Shah D., Ali M., Shukla D., Jain S., Aakalu V.K. (2017). Effects of histatin-1 peptide on human corneal epithelial cells. PLoS One.

[52] Lei X., Cheng L., Lin H., Pang M., Yao Z., Chen C., Forouzanfar T., Bikker F.J., Wu G., Cheng B. (2020). Human Salivary Histatin-1 Is More Efficacious in Promoting Acute Skin Wound Healing Than Acellular Dermal Matrix Paste. Front. Bioeng. Biotechnol..

[53] Oydanich M., Epstein S.P., Gadaria-Rathod N., Guers J.J., Fernandez K.B., Asbell P.A. (2018). In Vivo Efficacy of Histatin-1 in a Rabbit Animal Model. Curr. Eye Res..

[54] Liu Z., Du Y., Xu S., Li M., Lu X., Tian G., Ye J., Zhao B., Wei P., Wang Y. (2023). Histatin 1-modified SIS hydrogels enhance the sealing of peri-implant mucosa to prevent peri-implantitis. iScience.

[55] van Dijk I.A., Beker A.F., Jellema W., Nazmi K., Wu G., Wismeijer D., Krawczyk P.M., Bolscher J.G.M., Veerman E.C.I., Stap J. (2017). Histatin 1 Enhances Cell Adhesion to Titanium in an Implant Integration Model. J. Dent. Res..

[56] Pan L., Zhang X., Gao Q. (2022). Histatin-1 alleviates high-glucose injury to skin keratinocytes through MAPK signaling pathway. J. Cosmet. Dermatol..

[57] Ma D., Sun W., Fu C., Nazmi K., Veerman E.C.I., Jaspers R.T., Bolscher J.G.M., Bikker F.J., Wu G. (2022). GPCR/endocytosis/ERK signaling/S2R is involved in the regulation of the internalization, mitochondria-targeting and -activating properties of human salivary histatin 1. Int. J. Oral Sci..

[58] Son K.N., Lee H., Shah D., Kalmodia S., Miller R.C., Ali M., Balasubramaniam A., Cologna S.M., Kong H., Shukla D., Aakalu V.K. (2021). Histatin-1 is an endogenous ligand of the sigma-2 receptor. FEBS J..

[59] Huang G.Q., Yi G.G., Wu L.W., Feng S.F., Wu W., Peng L., Yi R.W., Ma W., Lu X. (2018). Protective effect of histatin 1 against ultraviolet-induced damage to human corneal epithelial cells. Exp. Ther. Med..

[60] Lei X.X., Cheng L.H.H., Lin H.Y., Yang Y., Lu Y.Y., Pang M.R., Dong Y.Q., Bikker F.J., Forouzanfar T., Cheng B., Wu G. (2022). The minimal active domain of human salivary histatin 1 is efficacious in promoting acute skin wound healing. Mil. Med. Res..

[61] Castro M., Torres P., Solano L., Córdova L.A., Torres V.A. (2019). Histatin-1 counteracts the cytotoxic and antimigratory effects of zoledronic acid in endothelial and osteoblast-like cells. J. Periodontol..

[62] Torres P., Hernández N., Mateluna C., Silva P., Reyes M., Solano L., Venegas S., Criollo A., Nazmi K., Bikker F.J. (2021). Histatin-1 is a novel osteogenic factor that promotes bone cell adhesion, migration, and differentiation. J. Tissue Eng. Regen. Med..

[63] Sun W., Ma D., Bolscher J.G.M., Nazmi K., Veerman E.C.I., Bikker F.J., Sun P., Lin H., Wu G. (2020). Human Salivary Histatin-1 Promotes Osteogenic Cell Spreading on Both Bio-Inert Substrates and Titanium SLA Surfaces. Front. Bioeng. Biotechnol..

[64] Sun W., Shi A., Ma D., Bolscher J.G.M., Nazmi K., Veerman E.C.I., Bikker F.J., Lin H., Wu G. (2020). All-trans retinoic acid and human salivary histatin-1 promote the spreading and osteogenic activities of pre-osteoblasts in vitro. FEBS Open Bio.

[65] Siwakul P., Sirinnaphakorn L., Suwanprateep J., Hayakawa T., Pugdee K. (2021). Cellular responses of histatin-derived peptides immobilized titanium surface using a tresyl chloride-activated method. Dent. Mater. J..

[66] Shi C., Yao Y., Wang L., Sun P., Feng J., Wu G. (2021). Human Salivary Histatin-1-Functionalized Gelatin Methacrylate Hydrogels Promote the Regeneration of Cartilage and Subchondral Bone in Temporomandibular Joints. Pharmaceuticals.

[67] Du Y., Chen M., Jiang J., Wang L., Wu G., Feng J. (2023). Hst1/Gel-MA Scaffold Significantly Promotes the Quality of Osteochondral Regeneration in the Temporomandibular Joint. J. Funct. Biomater..

[68] Arab A., Aghila Rani K.G., Altell R.T., Ismail A.A., Alkawas S., Samsudin A.R. (2022). The efficacy of salivary Histatin-1 protein in wound closure of nicotine treated human periodontal ligament fibroblast cells - In vitro study. Arch. Oral Biol..

[69] Cheng L., Lei X., Yang Z., Kong Y., Xu P., Peng S., Wang J., Chen C., Dong Y., Hu X. (2021). Histatin 1 enhanced the speed and quality of wound healing through regulating the behaviour of fibroblast. Cell Prolif..

[70] Wu A., Pathak J.L., Li X., Cao W., Zhong W., Zhu M., Wu Q., Chen W., Han Q., Jiang S. (2023). Human Salivary Histatin-1 Attenuates Osteoarthritis through Promoting M1/M2 Macrophage Transition. Pharmaceutics.

[71] Lee S.M., Son K.N., Shah D., Ali M., Balasubramaniam A., Shukla D., Aakalu V.K. (2021). Histatin-1 Attenuates LPS-Induced Inflammatory Signaling in RAW264.7 Macrophages. Int. J. Mol. Sci..

[72] Wang D., Wang H., Yan Y., Wei N., Jaspers R.T., Cao W., Lei X., Li S., Qi Y., Hu F. (2023). Coating 3D-Printed Bioceramics with Histatin Promotes Adhesion and Osteogenesis of Stem Cells. Tissue Eng. C Methods.

[73] Oudhoff M.J., Kroeze K.L., Nazmi K., van den Keijbus P.A.M., van 't Hof W., Fernandez-Borja M., Hordijk P.L., Gibbs S., Bolscher J.G.M., Veerman E.C.I. (2009). Structure-activity analysis of histatin, a potent wound healing peptide from human saliva: cyclization of histatin potentiates molar activity 1,000-fold. Faseb. J..

[74] Boink M.A., van den Broek L.J., Roffel S., Nazmi K., Bolscher J.G.M., Gefen A., Veerman E.C.I., Gibbs S. (2016). Different wound healing properties of dermis, adipose, and gingiva mesenchymal stromal cells. Wound Repair Regen..

[75] Imamura Y., Fujigaki Y., Oomori Y., Usui S., Wang P.L. (2009). Cooperation of salivary protein histatin 3 with heat shock cognate protein 70 relative to the G1/S transition in human gingival fibroblasts. J. Biol. Chem..

[76] Murakami Y., Nagata H., Shizukuishi S., Nakashima K., Okawa T., Takigawa M., Tsunemitsu A. (1994). Histatin as a synergistic stimulator with epidermal growth factor of rabbit chondrocyte proliferation. Biochem. Biophys. Res. Commun..

[77] Shah D., Son K.N., Kalmodia S., Lee B.S., Ali M., Balasubramaniam A., Shukla D., Aakalu V.K. (2020). Wound Healing Properties of Histatin-5 and Identification of a Functional Domain Required for Histatin-5-Induced Cell Migration. Mol. Ther. Methods Clin. Dev..

[78] Makihira S., Nikawa H., Shuto T., Nishimura M., Mine Y., Tsuji K., Okamoto K., Sakai Y., Sakai M., Imari N. (2011). Evaluation of trabecular bone formation in a canine model surrounding a dental implant fixture immobilized with an antimicrobial peptide derived from histatin. J. Mater. Sci. Mater. Med..

[79] Zhao Z., Sun X., Tu P., Ma Y., Guo Y., Zhang Y., Liu M., Wang L., Chen X., Si L. (2024). Mechanisms of vascular invasion after cartilage injury and potential engineering cartilage treatment strategies. Faseb. J..

[80] Wang X., Bove A.M., Simone G., Ma B. (2020). Molecular Bases of VEGFR-2-Mediated Physiological Function and Pathological Role. Front. Cell Dev. Biol..

[81] Ma D., Sun W., Nazmi K., Veerman E.C.I., Bikker F.J., Jaspers R.T., Bolscher J.G.M., Wu G. (2020). Salivary Histatin 1 and 2 Are Targeted to Mitochondria and Endoplasmic Reticulum in Human Cells. Cells.

[82] Han C., Barakat M., DiPietro L.A. (2022). Angiogenesis in Wound Repair: Too Much of a Good Thing?. Cold Spring Harbor Perspect. Biol..

